# A Case of Kaposi Sarcoma Misdiagnosed for 2 years: A Case Report

**DOI:** 10.1155/carm/5112520

**Published:** 2025-09-22

**Authors:** Maram T. Alkhatieb, Hussain A. Alkhalifah, Lama A. Aljilani, Saeed A. Alhudaifi, Doaa Y. Alqaidy

**Affiliations:** ^1^Department of Surgery, Faculty of Medicine, King Abdulaziz University, Jeddah, Saudi Arabia; ^2^Faculty of Medicine, King Abdulaziz University, Jeddah, Saudi Arabia; ^3^Department of Pathology, Faculty of Medicine, King Abdulaziz University, Jeddah, Saudi Arabia

**Keywords:** cellulitis, chronic venous ulcer, histopathology, HIV, Kaposi Sarcoma

## Abstract

**Background:** Kaposi Sarcoma (KS) is a rare low-grade vascular neoplasm that is associated with Human herpesvirus 8 (HHV-8). KS commonly affects HIV or immunocompromised patients. However, classical KS can be present even in the absence of these factors. In this case report, we describe a case of KS in an 83-year-old, HIV-negative Saudi male who was misdiagnosed for two years.

**Case Presentation:** The patient presented with right foot pain and swelling with two large masses. The patient was initially diagnosed with stasis dermatitis and chronic venous ulcer due to venous insufficiency and treated with endovenous thermal ablation. However, the patient's symptoms did not resolve, and he experienced several episodes of cellulitis that required multiple hospital admissions over a 2-year period. When the patient presented to our center, a biopsy was taken from the lesion, and it confirmed the diagnosis of KS.

**Conclusion:** KS or other underlying etiologies should be suspected in cases of recurrent unresolved infections, particularly in older patients. A high index of suspicion and a low threshold for biopsy are recommended to prevent missed or delayed diagnoses, which could ultimately lead to the worst prognosis.

## 1. Introduction

Kaposi Sarcoma (KS) is a rare low-grade vascular neoplasm with variably aggressive behavior depending on the clinical subtype. While it may behave indolently, particularly in classical cases, it can also exhibit locally aggressive or disseminated features in immunocompromised individuals [1, 2]. It is characterized by antiproliferative inflammatory changes of spindle cells in the endothelium of blood vessels [3, 4]. Human herpesvirus 8 (HHV-8) plays a major role in the pathogenesis of KS. The prevalence of HHV-8 in the Mediterranean region and Middle Eastern countries ranges between 10% and 20%, with significantly higher rates reported in sub-Saharan Africa [5, 6]. However, HHV-8 infection alone is insufficient to cause KS; a reduction in host immune surveillance—whether due to HIV, immunosuppressive therapy, or age-related immune decline—is typically required for disease progression [7]. KS most commonly presents with cutaneous lesions but may also involve visceral organs and lymph nodes, depending on the subtype.

There are four identified types of KS. Classical KS mostly affects men who are older than 50 years of Mediterranean, Eastern European, and Middle Eastern origin and has a male-to-female ratio of 17:1. Iatrogenic KS affects those using immunosuppressive drugs and transplant patients. African or endemic KS affects children in sub-Saharan Africa. And finally, endemic or acquired immunodeficiency syndrome (AIDS) KS, which is the most prevalent subtype [8, 9].

According to global cancer incidence data from 2022, KS affects approximately 0.5 per 100,000 males and 0.3 per 100,000 females annually [10]. Among patients living with HIV, the incidence per 100,000 person-years is substantially higher, reaching 280 in South Africa, 244 in Latin America, 237 in North America, 180 in Europe, and 52 in the Asia-Pacific region [11]. However, age has been shown to be an important risk factor for KS, as the incidence of KS increases with age [12].

In this case report, we present a case of foot KS in a Middle Eastern male who was misdiagnosed for 2 years. This case highlights the diagnostic delay that may occur when clinicians overlook KS in the differential diagnosis of chronic leg ulcers in elderly patients.

## 2. Case Presentation

An 83-year-old Saudi male patient, immunocompetent and HIV-negative, with no significant past medical history, and with no known Mediterranean or Eastern European ancestry, presented with a chronic history of unilateral right leg swelling extending from the foot to above the ankle. This was associated with skin erythema, while dorsalis pedis and posterior tibial pulses were palpable bilaterally. Initially, the patient was managed by dermatology as a case of stasis dermatitis with an antibiotic-based cream and salicylic acid ointments, followed by a 6-month follow-up. However, despite management, the symptoms did not resolve and progressively worsened. The swelling increased to the level of the knee, accompanied by localized warmth over the erythematous skin of the right leg. Additionally, a small ulcer formed on the dorsum of the ankle joint of the right foot. Subsequently, the patient was referred to a tertiary care center for further evaluation by vascular surgery to rule out any vascular pathology. Ultrasound imaging revealed significant dilation and reflux of the great saphenous vein (GSV), indicating GSV insufficiency. The patient was diagnosed with stasis dermatitis with chronic venous ulcer in the right foot and underwent endovenous thermal ablation of the right GSV for varicose veins, followed by conservative management that included therapeutic compression stockings, daily foot ulcer dressing, and regular follow-up with the vascular team. Despite these interventions, after more than 4 months, the symptoms continued to deteriorate, and the ulcer increased in size, with hyperpigmented skin developing over time. A fluid-filled bullous formation emerged, primarily over the ankle, dorsum, and plantar surface of the midfoot. These bullae subsequently ruptured, leading to mal-healing ulcers characterized by granulation tissue and fungating eschars.

**Figure 1:**
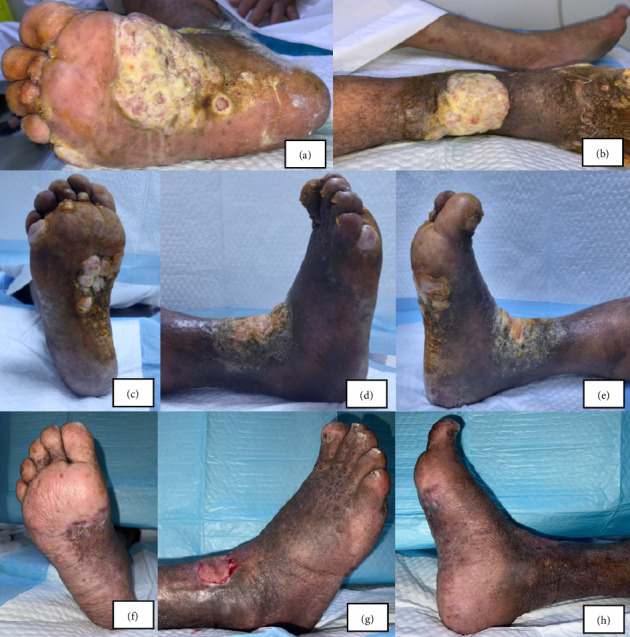
Clinical progression of the patient's right foot lesion. (a, b) The appearance of the plantar (a) and dorsum (b) surfaces of the right foot and lower leg at the time of presentation. (c–e) The same areas after 2 months of treatment, showing a partial response with reduced lesion size but persistence of ulceration. (f–h) The clinical status at 6 months into treatment, with marked improvement, healing of ulcerations, and flattening of lesions.

Following that, the patient had three admissions to the emergency department (ED) at another facility over several months due to episodes of altered consciousness and high fever, accompanied by similar right leg symptoms. The emergency team managed him as a case of right leg cellulitis, administering broad-spectrum oral antibiotics. During the third ED visit, the patient presented with severe leg pain and vital instability, necessitating intravenous (IV) antibiotics and admission to the intensive care unit (ICU) for close monitoring. The patient was subsequently discharged in stable condition, continuing treatment with mometasone and fusidic acid topical creams, along with follow-up in the outpatient department (OPD) with dermatology. Despite extensive treatment and ongoing symptoms, the definitive diagnosis remained unclear. After 2 years of unresolved issues, the patient presented to our tertiary care center's ED in a semiconscious state, complaining of needle-like pain in the right foot and swelling of the right lower limb. He was admitted for further investigation. Examination revealed unilateral right leg swelling with an inverted champagne bottle sign, accompanied by hyperpigmentation indicative of chronic venous insufficiency, lymphatic stippling consistent with lymphedema, fluid-filled bullous formation with foul smell, mall-healing ulcers with granulation tissues, and two fungating exophytic lesions were present, one in the dorsum of the foot against the ankle and the second in the middle of the plantar aspect of the foot, which suggested the presence of stasis dermatitis (Figure 1).

**Figure 2:**
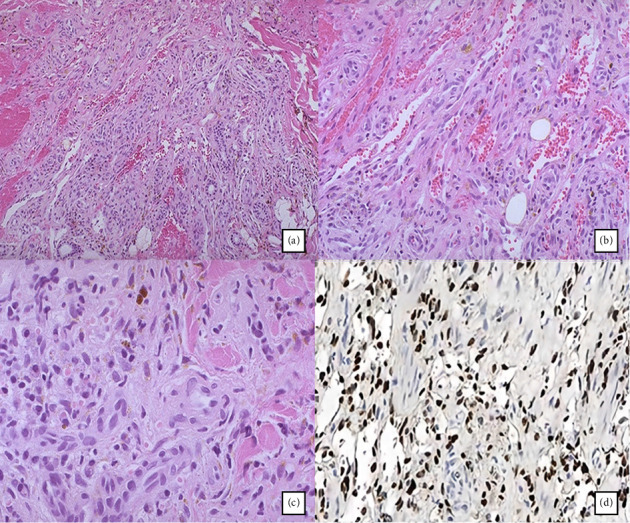
The histopathologic microscopic finding of the skin biopsy taken. (a) The neoplasm is composed of sheets of uniform spindle to epithelioid cells with mild pleomorphism. (b) A prominent congested blood vessels and extravasation of red blood cells are noted. (c) Some of the neoplastic cells have an epithelioid morphology. (d) The neoplastic cells are uniformly positive for HHV-8 immunostain.

The patient was admitted under the internal medicine team as a case of right foot cellulitis. Culture swaps were taken, and the patient was started on empirical antibiotic therapy and was scheduled to follow up in OPD with the infectious diseases regarding the culture results and the general surgery to take a skin biopsy of the lesion. A punch biopsy was performed at multiple sites of the lesions on the patient's right foot, revealing an infiltrative dermal-based lesion consisting of sheets of homogeneous spindle to epithelioid cells with minimal pleomorphism and well-defined slit-like vascular channels (Figure 2). Congested blood vessels and extravasation of erythrocytes are noted along with hemosiderophages and intra- and extracellular hyaline globules seen in Figure 2.

To further characterize the lesion, a panel of immunohistochemical studies is performed to reveal that neoplastic cells are positive for HHV-8, CD31, and CD34. Subsequently, an HIV test was conducted for the patient, yielding a negative result, and a diagnosis of KS was established.

The patient was referred to the oncology department, where CT scans of the chest, abdomen, and pelvis were done to rule out any metastasis. The imaging showed that the patient had no solid organ metastasis but showed metastatic disease to the right groin area as well as the right pelvic sidewall lymph nodes. The patient was set for palliative treatment by chemoradiation therapy without surgical intervention. The patient was put on Doxorubicin weekly, 25 mg/m^2^, and underwent 20 Gy of radiation in 5 fractions directed to the right foot in addition to daily wound dressing. The patient tolerated the treatment well and showed a significant decrease in the lesion sizes following treatment initiation (Figure 1).

## 3. Discussion

KS is a rare low-grade vascular neoplasm, accounting for approximately 1% of all cancers diagnosed globally [13]. Additionally, its association with HIV/AIDS further limits its prevalence, particularly in countries with low HIV rates, such as Saudi Arabia, where the prevalence of HIV is only 2 in 10,000 cases, according to the Saudi Ministry of Health [14]. Diagnosing KS in HIV-negative patients can be particularly challenging due to overlapping clinical features with more common conditions like cellulitis in acute presentations and vascular conditions such as stasis dermatitis or chronic venous ulcers in chronic cases [15–17].

In the early stages, KS skin lesions may resemble the reddened, swollen appearance typical of cellulitis. The overlapping symptoms, skin discoloration, swelling, pain, and warmth, often contribute to misdiagnosis as cellulitis. Without a definitive skin biopsy, KS may continue to be mistaken for cellulitis, especially in the absence of an HIV association. In this case, the patient also had lymph node metastasis, potentially leading to lymphedema, which increases the risk of local skin infections and further complicates the clinical picture [18]. In addition, the presence of venous insufficiency on the same side as the KS influenced the treating vascular surgery team to treat the patient as a case of stasis dermatitis with chronic venous ulceration, delaying the decision to perform a biopsy. When the patient first presented to our center with a prolonged history of a nonhealing chronic venous ulcer, malignancy was highly suspected at first sight. However, the initial suspicion was for squamous cell carcinoma (SCC) rather than KS, as SCC is the most common malignancy associated with chronic venous ulcers, and there was no history of HIV [19, 20]. However, KS may also present without any cutaneous manifestations—such as isolated visceral or nodal disease—so clinicians should consider it even when skin lesions are absent. Syrmos et al. reported a case of spinal KS without any skin involvement, underscoring the need to maintain a high index of suspicion for atypical presentations [21].

This case highlights the importance of early tissue diagnosis in atypical or nonhealing lesions. If a presumed benign lesion does not improve with appropriate standard therapy, clinicians should reconsider the diagnosis and promptly pursue a biopsy. In the context of cellulitis, for example, a lack of response to antibiotics within a few days strongly suggests an alternative diagnosis. However, KS lesions have been misdiagnosed as a variety of conditions, including not only cellulitis and stasis ulcers but even diabetic foot ulcers. There has been a reported case of classic KS in an HIV-negative diabetic patient whose foot lesion was initially thought to be a diabetic ulcer; the wound failed to heal with standard care, prompting a biopsy that revealed KS [22]. Such examples underscore that clinicians should maintain a high index of suspicion and a low threshold for biopsy of suspicious, atypical lesions that do not follow the expected healing course [23].

The etiopathogenesis of KS is caused by multiple factors, and there is established evidence linking it to HIV infection. Epidemiological investigations have demonstrated that the concomitant presence of HHV-8, the virus associated with KS, promotes angiogenesis, inflammation, and cell division while inhibiting apoptosis [8, 24, 25]. Other predisposing factors in classic (HIV-negative) KS include older age, male gender, chronic immune activation with elevated cytokines, genetic predisposition, and any form of immunosuppression [8]. In our patient, the absence of HIV suggests this is a case of classic KS, likely facilitated by HHV-8 infection alongside the patient's other risk factors (possibly including age or subtle immune dysregulation). Notably, our patient had an unusual disease course with regional lymph node involvement. Classic KS typically presents with indolent lesions on the extremities, and only 9%–15% of cases show trunk or visceral involvement; lymph node metastasis in classic KS is very rare. The presence of right groin lymph node metastasis in this case indicates a more aggressive behavior than is generally expected for classic KS, underlining the heterogeneity of this disease's presentation [26–28].

Treatment for KS depends on the number of lesions, type of lesions, and extent of the tumor, as well as the patient's overall condition [29]. In KS, the treatment goal is to relieve the symptoms, prevent progression, and reduce the edema [30]. However, there is no definitive treatment for KS [8]. Currently, in widespread usage, radiation therapy has been shown to be successful in treating individuals with local disease symptoms [22]. Due to the patient's disease being localized and symptomatic and his medical history, our institution's Medical Oncology Department, Radiation Oncology Department, and Multidisciplinary Tumor Board recommended local therapy. Local treatment options include chemotherapy, topical retinoid treatment, cryotherapy, photodynamic therapy, intralesional curettage, and electrodessication [31]. In our case, the patient was started with Doxorubicin weekly at 25 mg/m^2^. Usually, this drug is the first-line treatment, and it has been shown to reduce the size of lesions by greater than 50% of the original size in 71%–100% of cases [32, 33]. Furthermore, studies reported that it improves the typical KS hyperpigmentation with a response rate of 30%–60% [34]. Additionally, the patient underwent 20 Gy of radiation in 5 fractions without surgical intervention.

The patient's response to treatment was notably positive. After 2 months of therapy, he demonstrated good tolerance to the chemotherapy with no side effects, and there was a significant reduction in the size and number of lesions. The associated edema and pain also improved markedly. This outcome reinforces that, in the absence of a curative treatment for KS, a judiciously chosen combination of therapies can achieve disease control and symptom relief. It also underscores the importance of early diagnosis and intervention. Once the correct diagnosis of KS was established, appropriate therapy led to rapid improvement.

## 4. Conclusion

This case highlights the diagnostic challenge of KS in immunocompetent, HIV-negative individuals, particularly when clinical findings overlap with common vascular or infectious dermatologic conditions. In elderly patients with chronic, nonhealing lower limb lesions that are unresponsive to conventional management, malignancy should always remain a differential consideration. A high index of suspicion and a low threshold for biopsy are recommended to prevent missed or delayed diagnoses, as delayed diagnosis can lead to disease progression, lymph node involvement, and poorer outcomes.

Our patient benefited from palliative chemoradiation with significant clinical improvement, demonstrating that even advanced KS can respond well to nonsurgical management in appropriately selected patients. This case underscores the importance of multidisciplinary collaboration and vigilance in atypical presentations. Future efforts should aim to increase clinical awareness among frontline physicians, dermatologists, and vascular surgeons about the broad spectrum of KS presentations, even in the absence of traditional risk factors.

## Data Availability

All data used during this study are included in this published article and its figures.
